# Assessing Communication Skills of Medical Students in Objective Structured Clinical Examinations (OSCE) - A Systematic Review of Rating Scales

**DOI:** 10.1371/journal.pone.0152717

**Published:** 2016-03-31

**Authors:** Musa Cömert, Jördis Maria Zill, Eva Christalle, Jörg Dirmaier, Martin Härter, Isabelle Scholl

**Affiliations:** Department of Medical Psychology, University Medical Center Hamburg-Eppendorf, Hamburg, Germany; Cardiff University, UNITED KINGDOM

## Abstract

**Background:**

Teaching and assessment of communication skills have become essential in medical education. The Objective Structured Clinical Examination (OSCE) has been found as an appropriate means to assess communication skills within medical education. Studies have demonstrated the importance of a valid assessment of medical students’ communication skills. Yet, the validity of the performance scores depends fundamentally on the quality of the rating scales used in an OSCE. Thus, this systematic review aimed at providing an overview of existing rating scales, describing their underlying definition of communication skills, determining the methodological quality of psychometric studies and the quality of psychometric properties of the identified rating scales.

**Methods:**

We conducted a systematic review to identify psychometrically tested rating scales, which have been applied in OSCE settings to assess communication skills of medical students. Our search strategy comprised three databases (EMBASE, PsycINFO, and PubMed), reference tracking and consultation of experts. We included studies that reported psychometric properties of communication skills assessment rating scales used in OSCEs by examiners only. The methodological quality of included studies was assessed using the COnsensus based Standards for the selection of health status Measurement INstruments (COSMIN) checklist. The quality of psychometric properties was evaluated using the quality criteria of Terwee and colleagues.

**Results:**

Data of twelve studies reporting on eight rating scales on communication skills assessment in OSCEs were included. Five of eight rating scales were explicitly developed based on a specific definition of communication skills. The methodological quality of studies was mainly poor. The psychometric quality of the eight rating scales was mainly intermediate.

**Discussion:**

Our results reveal that future psychometric evaluation studies focusing on improving the methodological quality are needed in order to yield psychometrically sound results of the OSCEs assessing communication skills. This is especially important given that most OSCE rating scales are used for summative assessment, and thus have an impact on medical students’ academic success.

## Introduction

In the 21st century, teaching and assessment of communication skills in medical schools are well recognized [1]. Effective communication is considered to be one of the most important skills of a physician [2]. According to the Accreditation Council for Graduate Medical Education (ACGME), the American Board of Medical Specialties (ABMS), the Association of American Medical Colleges (AAMC), the General Medical Council (GMC), and the World Federation for Medical Education (WFME) communication and interpersonal skills are among the essential competencies to be taught in medical and residency programs [3–7]. Over the years, several international consensus statements have been published, which aim to provide educators with knowledge in development, implementation and evaluation of communication-oriented medical curricula [8–11].

Despite increasing significance of communication skills training in a medical setting, there is a lack of a generally accepted definition of adequate physician-patient communication [12]. Based on five widely recognized physician-patient communication models, the Kalamazoo I Consensus Statement extracted a list of the following seven key elements that characterize adequate physician-patient communication: a) building relationship, b) opening discussion, c) gathering information, d) understanding the patient’s perspective, e) sharing information, f) reaching agreement, and g) providing closure [9]. In addition, they represent a blueprint for the development of medical curricula comprising communication skills training and the assessment of students’ performance [13,14]. Empirical studies have demonstrated the importance of a valid assessment of medical students’ communication skills performance for several reasons [15]. First, through performance assessment students become aware of the relevance of physician-patient communication and receive feedback on their performance and deficits. Second, it enables educators to identify those medical students with significant deficits and reveals existing weaknesses within the curricula. Furthermore, summative assessments such as high-stake examinations could result in the denial of graduation in case of not qualified students to prevent damage from future patients [16].

To assess communication skills, most medical schools established the Objective Structured Clinical Examination (OSCE) using interactions with standardized patients (SP) [17]. An OSCE consists of several stations with different tasks and aims to simulate real clinical encounters between physician and patient. At that point it is important to emphasize that different kinds of OSCEs exist. They differ in their purpose. While some OSCEs address the assessment of communication skills in an integrated way as part of other clinical tasks (e.g. history taking, physical examination) there are also OSCEs which exclusively focus on the assessment of communication skills [2]. For the purpose of rating a student’s communication skills performance during an OSCE different kinds of rating scales have been developed [18–20]. Yet, the validity of the performance scores of a student is fundamentally dependent of the quality of the rating scales in use [21]. Nevertheless, a clear overview of the existing rating scales and their methodological and psychometric quality has not been conducted so far. Hence, a systematic review is needed to a) to compare and evaluate the existing rating scales based on well-defined quality criteria, b) to facilitate the choice of an appropriate instrument depending on the respective purpose, and c) to illustrate the gaps and needs in research, such as initiating the development of new instruments.

Therefore, this systematic review of rating scales on communication skills assessment in OSCEs aims at 1) identifying existing psychometrically tested rating scales on communication skills assessment in OSCEs and describing their underlying definition of communication skills, 2) determining the quality of design, methods and reporting of studies that analyze psychometric properties of rating scales, and 3) evaluating the psychometric quality of the identified rating scales.

## Methods

### Search strategy

We started our systematic review by performing an electronic literature search in the data bases EMBASE, PsycINFO and PubMed. We included all articles published between January 1979, the year in which the first OSCE to assess medical students’ clinical competence was developed [22], and January 2, 2015. For this purpose, it was necessary to devise a specific search strategy for each of the three data bases based on a combination of different terms and keywords from the following four domains: (i) construct, (ii) context, (iii) measurement, and (iv) psychometric properties. In addition, we made use of the PubMed search filter developed by Terwee et al. [23] to facilitate the search process for studies on psychometric properties of rating scales. Based on our predefined inclusion and exclusion criteria, we limited each of the three specific search strategies to peer-reviewed publications, published in English or German. Furthermore, we also excluded studies in which communication skills were just reported as a subscale and thus did not allow the extraction of results related solely to this subscale. The applied inclusion and exclusion criteria are displayed in Table 1. The full electronic search strategy is displayed in S1 Appendix. As part of our search strategy, we also performed a secondary search which consisted of reference tracking of all included full texts and consultation of experts in the field of communication skills in health care.

**Table 1:** Inclusion and exclusion criteria.

***Inclusion criteria***		*Excluded full texts (n = 49)*
1	The article is published in a peer-reviewed journal	4
2	The language of the publication is English or German	1
3	The publication date is between 1979 and 2015	
4	The measured construct is communication skills	16
5	The examinee is a single medical student	4
6	The underlying rating scale is used in an OSCE with standardized patients	5
7	The underlying rating scale is exclusively used by examiner	3
8	The rating scale is exclusively focused on communication skills	4
9	The aim of the study is to test the psychometric properties of the underlying scale	12
***Exclusion criteria***		
1	Not retrievable due to incomplete reference	
2	Full text not available	

Empty space = no full text was excluded for this reason.

### Study selection

First, we imported all search results into reference management software (EndNote) and removed all existing duplicates. Second, two reviewers (JZ and MC) independently performed a title and abstract screening to double-check the identified records for possible inclusion. In a next step, the remaining full texts were independently assessed for eligibility by two reviewers (EC and MC) using the inclusion and exclusion criteria. In case of disagreement regarding inclusion decisions, a third reviewer (IS) was consulted to reach consensus and to make a final decision.

### Data extraction and quality assessments

Final data extraction sheets were developed after pilot testing and adjustment in discussion between two reviewers (IS and MC). Data extraction sheets contained both descriptive data and data to assess the quality of the included studies. The process of assessing the quality comprised two separate steps. As a first step, the quality of design, methods and reporting of the included studies on psychometric properties was assessed by applying the COnsensus-based Standards for the selection of health Measurement INstruments (COSMIN) checklist with 4-point scale [24–26]. The second step addressed the evaluation of the psychometric properties of the identified rating scales with the quality criteria developed by Terwee et al. [27]. The COSMIN checklist and the quality criteria for good psychometric properties developed by Terwee et al. are described below. To ensure consistency in the application of the COSMIN checklist and the quality criteria by Terwee et al., an independent double assessment (EC and MC) was performed for a random sample of 15% of included papers (i.e. two studies) at the start of data collection. Any eventual initial disagreements and ambiguities were resolved through discussion prior to extracting and rating data for the remaining 85% of studies. Finally, data extraction and quality assessment were conducted by one reviewer (MC).

#### Assessment of methodological quality

The COSMIN checklist was developed in a multidisciplinary, international Delphi study and serves as a standardized tool for assessing the methodological quality of studies on measurement properties [24,25]. The COSMIN checklist consists of twelve boxes of which nine contain assessment standards for the following measurement properties: internal consistency, reliability, measurement error, content validity, structural validity, hypotheses testing, cross‐cultural validity, criterion validity and responsiveness. In addition, according to the predetermined instructions for completing the COSMIN checklist, it is necessary to complete the IRT box if Item-Response-Theory methods were used in a study [26]. Furthermore, there are two boxes on interpretability and generalizability, which serve the purpose to extract descriptive data. The number of items of the boxes varies between five and eighteen. Each of these items can be scored on the 4-point scale as excellent (+++), good (++), fair (+), or poor (0) based on specific criteria. To obtain an overall score for a box, the lowest score of any item has to be taken, which is called the “worst score counts” method. While we performed data extraction and evaluation for each of the twelve COSMIN boxes, we omitted the presentation of the boxes interpretability and generalizability because they do not provide further information to our descriptive data extraction of the included studies. It should be mentioned that the COSMIN checklist was primarily developed to facilitate the assessment of the methodological quality of Health-Related Patient-Reported Outcomes (HR-PROs) [24]. Since this systematic review exclusively focuses on observer based rating scales to assess communication skills of medical students within an OSCE, some of the items of the COSMIN checklist were rated as “not applicable” (n/a).

#### Assessment of psychometric quality

The criteria developed by Terwee et al. [27] were used to assess the quality of the psychometric properties. They have been successfully applied in previous reviews [21,28,29], one of them also including observer measures [29]. The Terwee et al. criteria address the following properties: content validity, internal consistency, criterion validity, construct validity, reproducibility (agreement and reliability), responsiveness, floor and ceiling effects and interpretability. Each of those eight properties can be evaluated by one item as positive (+), intermediate (?), negative (-) or no information available (0).

## Results

### Literature search and study selection

**Fig 1:**
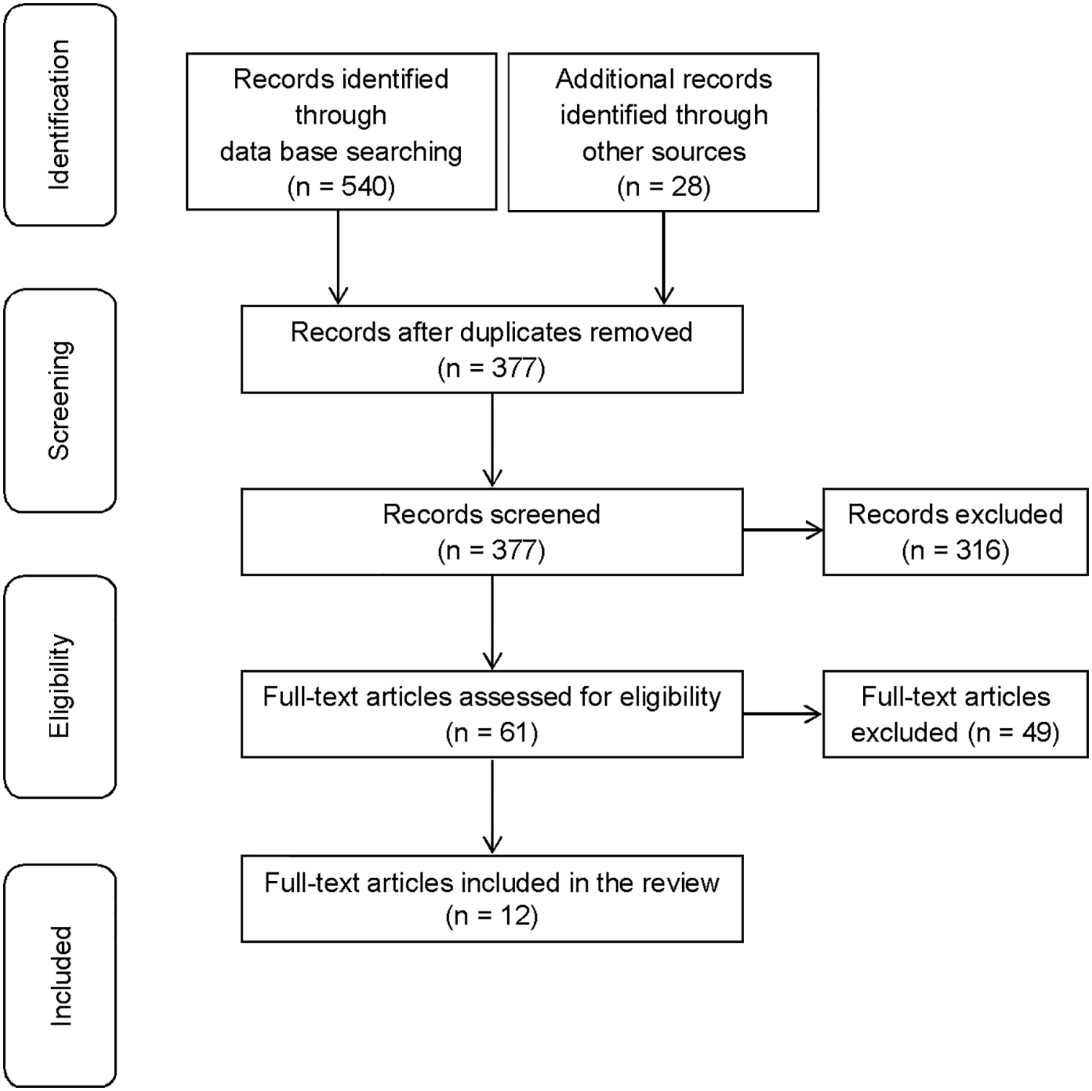
Flow diagram of study selection.

The electronic data base search yielded 540 records. In addition, 28 records were identified through secondary search of which 25 were from reference tracking and three from consultation of experts in the field of communication in health care. In a next step, 191 duplicates were removed. We then excluded another 316 records based on title and abstract screening. The full texts of the remaining 61 records were assessed for eligibility. Of the 61 records, 49 were excluded by applying the inclusion and exclusion criteria (see Table 1). As a result, twelve studies were included in this review. Most of the full texts were excluded either because the measured construct was not communication skills (n = 16) or the aim of the study was not to test the psychometric properties (n = 12). The study selection procedure is shown in Fig 1.

### Description of included studies and rating scales

The majority of the included studies were conducted in Europe. Of the twelve included studies reporting on eight rating scales, five were from UK [30–34], three from Germany [35–37], two from Canada [38,39] and one each from Belgium [40] and the US [41]. The study samples exclusively consisted of undergraduate medical students, with two of the studies being carried out during clinical clerkship [38,39]. Seven studies were initial studies with the objective of examining psychometric properties of a new measure [31–35,39,41]. The other five studies were further examinations of previously developed rating scales and reporting on their psychometric properties [30, 36–38,40]. Looking at the setting of the studies, it is important to underline that the OSCEs differed in their purpose between formative and summative evaluations. While formative OSCEs provide the examinee with performance feedback, summative OSCEs enable the examiners to make pass-fail decisions based on predefined criteria [42,43]. Most of the OSCEs in our systematic review were exclusively used for summative evaluations [30,31, 33, 34,36,38,40]. Descriptive data of the included studies are displayed in Table 2.

**Table 2:** Descriptive data of the included studies.

Measure	Authors (Year)	Setting*	Study sample*	Country
**EPSCALE**	Silverman et al. (2011)	summative OSCE with UMS during final MB examinations at University of Cambridge	n = 124**	UK
**EPSCALE**	Edgcumbe et al. (2012)	summative OSCE with UMS during final MB examinations at University of Cambridge	n = 124**	UK
**MCS-OSCE**	Fischbeck et al. (2011)*	formative and summative OSCE with UMS attending a Medical Psychology and Medical Sociology course at Johannes Gutenberg University Mainz	sample 1: n = 182, 61% f, m_age_ = 22 ys; sample 2: n = 181, 66% f, m_age_ = 22 ys	Germany
**CCAT**	Harasym et al. (2008)	summative OSCE with UMS during clinical clerkship at University of Calgary	n = 190	Canada
**AG-OSCE-R**	Hodges & McIlroy (2003)	formative OSCE with UMS during clinical clerkship in 3rd or 4th study year at University of Toronto	n = 57	Canada
**AG-OSCE-R**	Scheffer et al. (2008)	formative OSCE with UMS in 2nd or 3rd study year at Charité-Universitätsmedizin Berlin	n = 113, 65% f, m_age_ = 23 ys (SD_age_ = 4)	Germany
**AG-OSCE-R**	Mortsiefer et al. (2014)	summative OSCE with UMS in 4th study year at Heinrich-Heine-University Düsseldorf	n = 453	Germany
**LCSAS**	Humphris & Kaney (2001)*	summative OSCE with UMS in 1st or 2nd study year at University of Liverpool	sample 1: n = 600; sample 2: n = 60; sample 3: n = 80	UK
**LUCAS**	Huntley et al. (2012)*	formative and summative OSCE with UMS in 1st study year at University of Liverpool	sample 1: n = 731, 53.7% f, m_age_ = 19 ys (SD_age_ = 1.61); sample 2: n = 40	UK
**LIDM-RS**	Thistlethwaite (2002)	summative OSCE with UMS in 3rd study year at University of Leeds	n = 194	UK
**CG**	Lang et al. (2004)*	formative OSCE with UMS of two cohorts at East Tennessee State University and Tulane University	sample 1: n = 50; sample 2: n = 50	USA
**CG**	Van Nuland et al. (2012)	summative OSCE with UMS in their final undergraduate year at Catholic University of Leuven	n = 63	Belgium

*some studies were conducted in more than one setting and/or used more than one sample,

**Silverman et al. (2011) and Edgcumbe et al. (2012) used the same study sample, UMS = undergraduate medical students, MB = Medicinae Baccalaureus,

f = female, ys = years, m = mean, SD = standard deviation. Full titles of the rating scales: Explanation and Planning Scale (EPSCALE), Mayence Communication Skills OSCE (MCS-OSCE), Calgary-Cambridge Assessment Tool (CCAT), Analytic Global OSCE Rating (AG-OSCE-R), Liverpool Communication Skills Assessment Scale (LCSAS), Liverpool Undergraduate Communication Assessment Scale (LUCAS), Leeds Informed Decision Making Rating Scale (LIDM-RS), Common Ground (CG).

The present review included eight rating scales which have been applied in OSCE settings to assess communication skills of medical students while they interacted with SPs. From these eight rating scales, five were clearly named by the authors [30–33, 38,40,41]. For the remaining three rating scales we had come up with an acronym based on information from title or abstract. Thus, MCS-OSCE stands for the Mayence Communication Skills OSCE [35], AG-OSCE-R for the Analytic Global OSCE Rating [39] and finally LIDM-RS for the Leeds Informed Decision Making Rating Scale [34]. One of the three aims of this review was to describe the underlying definition of communication skills of the included rating scales. As displayed in Table 3, not all of the eight rating scales are explicitly developed based on a clear definition of communication skills. Of the eight rating scales, five includea definition of communication skills [30,32,33,35,38,40,41]. The underlying definition of two rating scales [30,33,38] is based on the Calgary-Cambridge Guide, which is a model for medical interview [44,45]. One measure [40,41] derives its definition of communication skills from the Toronto and Kalamazoo Consensus Statements [9, 10]. Finally, there are two rating scales that contain their own specific definition of communication skills [32,35]. Descriptive data of the included rating scales are shown in Table 3.

**Table 3:** Descriptive data of the included rating scales.

Measure	Authors (Year)	Underlying definition of communication skills	Language	Dimensions	Items	Response
**EPSCALE**	Silverman et al. (2011)	definition based on Calgary-Cambridge Guide	English	6	15	4-point scale
	Edgcumbe et al. (2012)	definition based on Calgary-Cambridge Guide	English	6	15	4-point scale
**MCS-OSCE**	Fischbeck et al. (2011)	own definition	German	n/r	n/r	5-point scale
**CCAT**	Harasym et al. (2008)	definition based on Calgary-Cambridge Guide	English	3	28	3-point scale
**AG-OSCE-R**	Hodges & McIlroy (2003)	n/r	English	4	4	5-point scale
	Scheffer et al. (2008)	n/r	German	4	4	5-point scale
	Mortsiefer et al. (2014)	n/r	German	4	4	5-point scale
LCSAS*	Humphris & Kaney (2001)	n/r	English	5	12	4-point scale
**LUCAS**	Huntley et al. (2012)	own definition	English	2	10	4 items using 2 response options**;
						6 items using 3 response options***
**LIDM-RS**	Thistlethwaite (2002)	n/r	English	n/r	10	3-point scale
**CG**	Lang et al. (2004)	definition based on Toronto and Kalamazoo Consensus Statements	English	7	36	mixed
	Van Nuland et al. (2012)	definition based on Toronto and Kalamazoo Consensus Statements	Dutch	7	7	7-point scale

*The LCSAS is just one part of the communication skills assessment system of Humphris & Kaney (2001). The LCSAS is intended to be applied with the Global Simulated Patient Rating Scale (GSPRS). We excluded the GSPRS since it is a rating scale used by SPs.

**The two response options were competent-unacceptable.

***The three response options were competent-borderline-unacceptable. n/r = not reported, cs = communication skills. Full titles of the rating scales: Explanation and Planning Scale (EPSCALE), Mayence Communication Skills OSCE (MCS-OSCE), Calgary-Cambridge Assessment Tool (CCAT), Analytic Global OSCE Rating (AG-OSCE-R), Liverpool Communication Skills Assessment Scale (LCSAS), Liverpool Undergraduate Communication Assessment Scale (LUCAS), Leeds Informed Decision Making Rating Scale (LIDM-RS), Common Ground (CG).

### Quality of design, methods and reporting

The assessment of the methodological quality of the included studies on measurement properties by applying the COSMIN checklist is presented in Table 4. None of the twelve studies reported on all of the nine COSMIN boxes. One of the twelve included studies used Item-Response-Theory [38]. Furthermore, another three studies applied the generalizability theory [46] to assess reliability (Box B) [31,33,40]. To assess these studies properly, it was necessary to make minor adjustments to the respective COSMIN box. Internal consistency (Box A) was calculated in seven studies [32,33,35,36,39–41]. Only one of them [32] received an excellent score, while the other six studies [33,35,36,39–41] were rated poor. Reliability (Box B) was addressed in ten studies [31–38,40,41]. Six of them scored excellent [31–33,36–38], one good [40], one fair [35] and two poor [34,41]. Measurement error (Box C) was not reported in any of the included studies. Of the seven studies [31–35,39,41], where content validity could be rated (Box D), only one study was rated fair [31], while the other six studies scored poorly [32–35,39,41]. Only two studies addressed the structural validity (Box E) [30,32] of which one scored excellent [32] and one good [30]. Hypotheses testing (Box F) was conducted in eight studies [31,32,35–37,39–41]. Two of them [32,35] were rated good, whereas the other six studies [31,36,37,39–41] received a poor score. One study [37] translated a measure into German and received a poor score regarding the translation procedure. Detailed results for COSMIN ratings on item level are shown in the S2 Appendix.

**Table 4:** Quality of design, methods and reporting of studies on psychometric properties.

*Measure*	*Authors (Year)*	*IRT Box*	*Psychometric properties*								
			A	B	C	D	E	F	G	H	I
**EPSCALE**	Silverman et al. (2011)		0	+++		0					
	Edgcumbe et al. (2012)						++				
**MCS-OSCE**	Fischbeck et al. (2011)		0	+^a^		0		++			
**CCAT**	Harasym et al. (2008)	+++		+++							
**AG-OSCE-R**	Hodges & McIlroy (2003)		0			0		0			
	Scheffer et al. (2008)			+++^a^				0	0^c^		
	Mortsiefer et al. (2014)		0	+++^a^				0			
**LCSAS**	Humphris & Kaney (2001)			+++^a^		+		0			
**LUCAS**	Huntley et al. (2012)		+++	+++^a^		0	+++	++			
**LIDM-RS**	Thistlethwaite (2002)			0^a^		0					
**CG**	Lang et al. (2004)		0	0^a^^,^^b^		0		0			
	Van Nuland et al. (2012)		0	++				0			

COSMIN psychometric property boxes: IRT Box = General requirements for studies that applied Item Response Theory (IRT) models, A = internal consistency, B = reliability, C = measurement error, D = content validity, E = structural validity, F = hypotheses testing, G = cross-cultural validity, H = criterion validity, I = responsiveness. 4-point scale rating: +++ = excellent, ++ = good, + = fair, 0 = poor, empty space = COSMIN rating not applicable. For exact information regarding the definitions of psychometric properties and 4-point scale rating see COSMIN website (www.cosmin.nl).

^a^ = Inter-rater-reliability,

^b^ = Intra-rater-reliability,

^c^ = only evaluation of the quality of the translation procedure.

### Quality of psychometric properties

The evaluation of the psychometric properties of the identified rating scales was carried out by applying the quality criteria of Terwee et al. The corresponding results are shown in Table 5. Content validity received negative scores in all of the seven respective studies [31–35,39,41]. In case of internal consistency a positive rating was received in one study [32] and an intermediate rating in six studies [31,32,39–41]. Construct validity was evaluated positively in three studies [35–37] and received intermediate rating in five studies [31,32,39–41]. None of the included studies provided any information on agreement, while ten studies contained information on reliability. Reliability was rated positively in three studies [32,37,38], intermediate in one study [41], and negative in six studies [31,33–36,40]. Regarding criterion validity, responsiveness and floor and ceiling effects, none of the studies gave any information. Finally, interpretability was reported in seven studies and was judged as intermediate in all of them [31,34–37,39,40].

**Table 5:** Quality of psychometric properties.

*Instruments / Authors / (Year)*	*Content validity*	*Internal consistency*	*Criterion validity*	*Construct validity*	*Reproducibility (Agreement)*	*Reproducibility (Reliability)*	*Responsiveness*	*Floor & ceiling effects*	*Interpretability*
**EPSCALE**	**-0**	**?0**	**00**	**00**	**00**	**-0**	**00**	**00**	**00**
Silverman et al. (2011)	-	?	0	0	0	-	0	0	0
Edgcumbe et al. (2012)	0	0	0	0	0	0	0	0	0
**MCS-OSCE**	**-**	**?**	**0**	**+**	**0**	**-**	**0**	**0**	**?**
Fischbeck et al. (2011)	-	?	0	+	0	-	0	0	?
**CCAT**	**0**	**0**	**0**	**0**	**0**	**+**	**0**	**0**	**0**
Harasym et al. (2008)	0	0	0	0	0	+	0	0	0
**AG-OSCE-R**	**-00**	**?0?**	**000**	**?++**	**000**	**0+-**	**000**	**000**	**???**
Hodges & McIlroy (2003)	-	?	0	?	0	0	0	0	?
Scheffer et al. (2008)	0	0	0	+	0	+	0	0	?
Mortsiefer et al. (2014)	0	?	0	+	0	-	0	0	?
**LCSAS**	**-**	**0**	**0**	**?**	**0**	**-**	**0**	**0**	**?**
Humphris & Kaney (2001)	-	0	0	?	0	-	0	0	?
**LUCAS**	**-**	**+**	**0**	**?**	**0**	**+**	**0**	**0**	**0**
Huntley et al. (2012)	-	+	0	?	0	+	0	0	0
**LIDM-RS**	**-**	**0**	**0**	**0**	**0**	**-**	**0**	**0**	**?**
Thistlethwaite (2002)	-	0	0	0	0	-	0	0	?
**CG**	**-0**	**??**	**00**	**??**	**00**	**?-**	**00**	**00**	**0?**
Lang et al. (2004)	**-**	?	0	?	0	?	0	0	0
Van Nuland et al. (2012)	0	?	0	?	0	-	0	0	?

Rating: + = positive, ? = intermediate,— = negative, 0 = no information available. Grey lines summarize ratings of psychometric properties per measure. For exact information regarding the definitions of psychometric properties see Terwee et al. [27].

## Discussion

The present systematic review aimed at identifying psychometrically tested rating scales on communication skills assessment in OSCEs, describing their underlying definition of communication skills, assessing the methodological quality of the included studies and evaluating the psychometric quality of the identified rating scales. For these purposes, data were extracted from twelve studies reporting on eight rating scales.

Regarding the underlying definition of communication skills of the identified rating scales, publications on three of the eight identified rating scales (AG-OSCE-R [36,37,39], LCSAS [31] and LIDM-RS [34]) did not provide any information on how communication skills were defined. This is certainly a shortcoming, as it would be important for readers of these papers to know on what basis items were developed, especially for educators, who might want to use these scales for OSCE assessment at their university. On the other hand, many of the rating scales (EPSCALE [30,33], CCAT [38] and CG [40,41]) are either based on the well-known model of Calgary-Cambridge Guide [44,45] or on the much-cited consensus statements of Toronto and Kalamazoo [9, 10]. In terms of using one of the identified rating scales in a specific medical education setting, we recommend checking whether a measure’s definition of communication skills matches the definition given in the curriculum of the specific setting.

The process of assessing the methodological quality of the included studies by applying the COSMIN checklist revealed that most studies were mainly poorly rated. One exception was the quality of the assessment of reliability, which was rated as excellent in most studies. Another main exception was the study reporting on psychometric properties of LUCAS [32], which received mainly excellent and good scores. However, its content validity was rated of poor quality. Another study worth mentioning positively was the one reporting on psychometric properties of the CCAT [38]. Although it only tested reliability by using the Item-Response-Theory, it was rated of excellent quality. When comparing the COSMIN ratings between studies, the measure development study of the CG [41] received the lowest ratings. All of four psychometric properties reported in this study were rated poor. Looking at the COSMIN ratings on the item level (see S2 Appendix), it is important to emphasize that they reveal a more differentiated picture. Several studies scored excellent or good on many items of the nine COSMIN boxes. However, under the terms of the “worst score counts” method of COSMIN to obtain an overall score for a box the lowest score of any item had to be taken, which led to poor psychometric property ratings for many studies. Thus, many studies could have performed much better in terms of methodological quality, if they would have taken into account the recommendations of the COSMIN group.

The evaluation of the psychometric properties using the criteria developed by Terwee et al. showed that the psychometric quality of the eight identified rating scales was mainly intermediate. The measure LUCAS [32] received the best rating in terms of psychometric quality. However, it is remarkable that none of the rating scales received a positive or an intermediate quality rating on content validity. Based on the fact that content validity is meant to be one of the most important psychometric properties [27], these assessments on content validity represent a major flaw.

The corresponding results of the methodological quality resulting from the COSMIN checklist and of the psychometric quality with the Terwee et al. criteria have to be taken into account together to draw conclusions appropriately. In this review several serious flaws concerning design, methods and reporting of the included studies could be shown by applying the COSMIN checklist. Thus, it is important to note that the results of the Terwee criteria on the psychometric quality of the rating scales need to be interpreted with care, as it is difficult to say how much one can trust the results gained from studies with poor design, methods and reporting. Combining the results of the COSMIN checklist and the Terwee et al. criteria, LUCAS [32] had the best results. Nevertheless, it must be underlined that its content validity is not satisfactory and should be checked in future research. It is also important to mention that some of the rating scales scored excellent or good on the methodical rating with COSMIN, while the evaluation with the Terwee et al. criteria clearly revealed poor psychometric properties. These results have a higher credibility than those gained from methodologically flawed studies.

Our systematic review has several strengths. First, we devised a specific search strategy for each of the three data bases in order to identify all records relevant to our purpose. Second, two reviewers independently performed a title and abstract screening to double-check the identified records for possible inclusion. Third, as recommended, the process of assessing the quality comprised two separated steps using the COSMIN checklist with 4-point scale rating to rate the methodological quality of the included studies and the quality criteria for good psychometric properties developed by Terwee et al. to determine the quality of the psychometric properties. The assessment of the methodological quality of the included studies is intended to make sure that psychometric properties reported in the studies can be interpreted and rated appropriately. Besides its strengths, this present review has also several limitations. First, our search was limited to English and German. Hence, it is possible that we might have failed to notice relevant publications. To minimize this risk, we also performed a secondary search which consisted of reference tracking of all included full texts and consultation of a range of international experts in the field of communication in health care. Second, 85% of the process of data extraction and quality assessment was performed by one reviewer only. Thus, it cannot be excluded with certainty that the assessment of included studies and psychometric quality of the identified rating scales were biased. However, a double assessment was performed for the first two studies in order to discuss and to resolve eventual initial ambiguities regarding the application of the COSMIN checklist and the Terwee et al. criteria. Third, due to our inclusion and exclusion criteria, we exclusively focused on rating scales used by examiners. Thus, we excluded rating scales that are meant to be completed by standardized patients to assess medical students’ communication skills. These tools can also be of high value, especially for formative assessment of communication skills and it might be interesting for future research to examine the performance of those measures as well.

In this systematic review eight rating scales assessing the communication skills of medical students in OSCE settings were identified. According to our results, the development of new rating scales is not necessarily required. Instead, efforts need to be made to eliminate the existing flaws. The COSMIN checklist illustrated several research gaps in the methodological quality of psychometric evaluation studies, which have to be approached. Since the methodological quality of the psychometric evaluation studies represents the basis for the evaluation of psychometric properties, it is indispensable to improve it. For this purpose, we recommend to use more rigorous methodological designs and a more detailed reporting. First, future psychometric studies need to conduct and describe the testing of content validity in more detail. Second, analyses of the factorial structure of the rating scales should be performed, which has an impact on internal consistency and structural validity. For hypotheses testing (on convergent or divergent validity) to be improved, future evaluation studies need clearly formulated hypotheses, larger sample sizes for multiple hypotheses and an adequate description of the comparator rating scales. Third, several psychometric properties (e.g. measurement error, floor and ceiling effects, responsiveness) were completely neglected in all included studies. Thus, they deserve attention in future psychometric evaluation studies.

## Conclusion

Our systematic review gives an overview of rating scales, which are applied within the medical education setting to assess students’ communication skills. It can help teachers and researchers in the field of medical education to find the appropriate measure for their individual purpose. Nevertheless, we identified several research gaps regarding the methodological quality of studies reporting on psychometric properties and the quality of their results. Based on our results, the use of the eight identified rating scales to assess students’ communication skills needs to be done with care, as their methodological quality is not completely satisfactory. Hence, future psychometric evaluation studies focusing on improving the methodological quality are needed in order to yield psychometrically sound results of the OSCEs assessing communication skills. This is especially important considering that most rating scales included in this review were used for summative evaluation, i.e. to make pass-fail decisions. Such decisions have a high impact on students’ academic success and should be based on reliable and valid assessment.

## Supporting Information

S1 Appendix Electronic data base search strategy for EMBASE, PsycINFO, PubMed. (DOCX)

S2 Appendix Detailed results for the COSMIN checklist. (DOCX)

S1 Checklist PRISMA checklist. (DOC)
