# Supplementary material for: Assessing Communication Skills of Medical Students in Objective Structured Clinical Examinations (OSCE) - A Systematic Review of Rating Scales
Source: PLoS One. 2016 Mar 31;11(3):e0152717. doi: 10.1371/journal.pone.0152717 (PMC4816391; doi:10.1371/journal.pone.0152717)
Supplement: S1 Appendix — (DOCX) [file pone.0152717.s001.docx]

# S1 Appendix. Electronic data base search strategy for EMBASE, PsycINFO, PubMed.

**EMBASE via Ovid**Communication (OR communication skill*/ communicative skill*/ interaction/ ability to communicate/ articulation/ communicative competence/ interpersonal relation*/ professional competence/ interpersonal skill*/ interpersonal communication/ interpersonal behavio?r/ verbal behavio?r)
AND
OSCE (OR Objective Structured Clinical Examination/ clinical skill performance)
AND
scale* (OR instrument*/ measur*/ checklist/ questionnair*/ assess*/ rating/ coding/ test/ evaluation)
AND
psychometric* (OR reliab*/ valid*/ accura*/ consisten*/ Cronbach*/ factor*/ internal consistency/ measurement error/ hypotheses testing/ cross-cultural validity/ responsiveness/ test-retest/ Item Response/ Rasch analysis/ factor* analy*is/ factor structure)

Limitations:

- 1979 - current
- English OR German

**PsycINFO via Ovid**
Communication (OR communication skill*/ communicative skill*/ interaction/ ability to communicate/ articulation/ communicative competence/ interpersonal relation*/ professional competence/ interpersonal skill*/ interpersonal communication/ interpersonal behavio?r/ verbal behavio?r)
AND
OSCE (OR Objective Structured Clinical Examination/ clinical skill performance)
AND
scale* (OR instrument*/ measur*/ checklist/ questionnair*/ assess*/ rating/ coding/ test/ evaluation)
AND
psychometric* (OR reliab*/ valid*/ accura*/ consisten*/ Cronbach*/ factor*/ internal consistency/ measurement error/ hypotheses testing/ cross-cultural validity/ responsiveness/ test-retest/ Item Response/ Rasch analysis/ factor* analy*is/ factor structure)

Limitations:

- 1979 - current
- English OR German

**PubMed with COSMIN filter**
Communication (OR “communication skill*”/ “communicative skill*”/ interaction/ “ability to communicate”/ articulation/ “communicative competence”/ “interpersonal relation*”/ “professional competence”/ “interpersonal skill*”/ “interpersonal communication”/ “interpersonal behavior”/ “verbal behavior”/ “interpersonal behaviour”/ “verbal behaviuor”) AND
OSCE (OR “Objective Structured Clinical Examination”/ “clinical skill performance”)
AND
scale* (OR instrument*/ measur*/ checklist/ questionnair*/ assess*/ rating/ coding/ test/ evaluation)
AND
(instrumentation[sh] OR Validation Studies[pt] OR “reproducibility of results”[MeSH Terms] OR reproducib*[tiab] OR “psychometrics”[MeSH] OR psychometr*[tiab] OR clinimetr*[tiab] OR clinometr*[tiab] OR “observer variation”[MeSH] OR observer variation[tiab] OR “discriminant analysis”[MeSH] OR reliab*[tiab] OR valid*[tiab] OR coefficient[tiab] OR “internal consistency”[tiab] OR (cronbach*[tiab] AND (alpha[tiab] OR alphas[tiab])) OR “item correlation”[tiab] OR “item correlations”[tiab] OR “item selection”[tiab] OR “item selections”[tiab] OR “item reduction”[tiab] OR “item reductions”[tiab] OR agreement[tw] OR precision[tw] OR imprecision[tw] OR “precise values”[tw] OR test–retest[tiab] OR (test[tiab] AND retest[tiab]) OR (reliab*[tiab] AND (test[tiab] OR retest[tiab])) OR stability[tiab] OR interrater[tiab] OR inter-rater[tiab] OR intrarater[tiab] OR intra-rater[tiab] OR intertester[tiab] OR inter-tester[tiab] OR intratester[tiab] OR intra-tester[tiab] OR interobserver[tiab] OR inter-observer[tiab] OR intraobserver[tiab] OR intra-observer[tiab] OR intertechnician[tiab] OR inter-technician[tiab] OR intratechnician[tiab] OR intra-technician[tiab] OR interexaminer[tiab] OR inter-examiner[tiab] OR intraexaminer[tiab] OR intra-examiner[tiab] OR interassay[tiab] OR inter-assay[tiab] OR intraassay[tiab] OR intra-assay[tiab] OR interindividual[tiab] OR inter-individual[tiab] OR intraindividual[tiab] OR intra-individual[tiab] OR interparticipant[tiab] OR inter-participant[tiab] OR intraparticipant[tiab] OR intra-participant[tiab] OR kappa[tiab] OR kappa’s[tiab] OR kappas[tiab] OR “coefficient of variation”[tiab] OR repeatab*[tw] OR ((replicab*[tw] OR repeated[tw]) AND (measure[tw] OR measures[tw] OR findings[tw] OR result[tw] OR results[tw] OR test[tw] OR tests[tw])) OR generaliza*[tiab] OR generalisa*[tiab] OR concordance[tiab] OR (intraclass[tiab] AND correlation*[tiab]) OR discriminative[tiab] OR “known group”[tiab] OR “factor analysis”[tiab] OR “factor analyses”[tiab] OR “factor structure”[tiab] OR “factor structures”[tiab] OR dimensionality[tiab] OR subscale*[tiab] OR “multitrait scaling analysis”[tiab] OR “multitrait scaling analyses”[tiab] OR “item discriminant”[tiab]OR “interscale correlation”[tiab] OR “interscale correlations”[tiab] OR ((error[tiab] OR errors[tiab]) AND (measure*[tiab] OR correlat*[tiab] OR evaluat*[tiab] OR accuracy[tiab] OR accurate[tiab] OR precision[tiab] OR mean[tiab])) OR “individual variability”[tiab] OR “interval variability”[tiab] OR “rate variability”[tiab] OR “variability analysis”[tiab] OR (uncertainty[tiab] AND (measurement[tiab] OR measuring[tiab])) OR “standard error of measurement”[tiab] OR sensitiv*[tiab] OR responsive*[tiab] OR (limit[tiab] AND detection[tiab]) OR “minimal detectable concentration”[tiab] OR interpretab*[tiab] OR (small*[tiab] AND (real[tiab] OR detectable[tiab]) AND (change[tiab] OR difference[tiab])) OR “meaningful change”[tiab] OR “minimal important change”[tiab] OR “minimal important difference”[tiab] OR “minimally important change”[tiab] OR “minimally important difference”[tiab] OR “minimal detectable change”[tiab] OR “minimal detectable difference”[tiab] OR “minimally detectable change”[tiab] OR “minimally detectable difference”[tiab] OR “minimal real change”[tiab] OR “minimal real difference”[tiab] OR “minimally real change”[tiab] OR “minimally real difference”[tiab] OR “ceiling effect”[tiab] OR “floor effect”[tiab] OR “Item response model”[tiab] OR IRT[tiab] OR Rasch[tiab] OR “Differential item functioning”[tiab] OR DIF[tiab] OR “computer adaptive testing”[tiab] OR “item bank”[tiab] OR “cross-cultural equivalence”[tiab])
NOT
(“addresses”[Publication Type] OR “biography”[Publication Type] OR “case reports”[Publication Type] OR “comment”[Publication Type] OR “directory”[Publication Type] OR “editorial”[Publication Type] OR “festschrift”[Publication Type] OR “interview”[Publication Type] OR “lectures”[Publication Type] OR “legal cases”[Publication Type] OR “legislation”[Publication Type] OR “letter”[Publication Type] OR “news”[Publication Type] OR “newspaper article”[Publication Type] OR “patient education handout”[Publication Type] OR “popular works”[Publication Type] OR “congresses”[Publication Type] OR “consensus development conference”[Publication Type] OR “consensus development conference, nih”[Publication Type] OR “practice guideline”[Publication Type]) NOT (“animals”[MeSH Terms] NOT “humans”[MeSH Terms])

Electronic data base searches were conducted on the 2^nd^ of January in 2015.

**COSMIN-Filter**

Terwee, C. B., Jansma, E. P., Riphagen, I. I., & de Vet, H. C. (2009). Development of a methodological PubMed search filter for finding studies on measurement properties of measurement instruments. Quality of Life Research, 18(8), 1115-1123.

Source: http://link.springer.com/article/10.1007%2Fs11136-009-9528-5/fulltext.html
