# Supplementary material for: Assessing Communication Skills of Medical Students in Objective Structured Clinical Examinations (OSCE) - A Systematic Review of Rating Scales
Source: PLoS One. 2016 Mar 31;11(3):e0152717. doi: 10.1371/journal.pone.0152717 (PMC4816391; doi:10.1371/journal.pone.0152717)
Supplement: S2 Appendix — (DOCX) [file pone.0152717.s002.docx]

# S2 Appendix. Detailed results for the COSMIN checklist.

| ***Box*** | ***COSMIN psychometric properties/Items**** | ***Study IDs/Rating scores*** | | | | | | | | | | | | | | | | | | | | | |
| --- | --- | --- | --- | --- | --- | --- | --- | --- | --- | --- | --- | --- | --- | --- | --- | --- | --- | --- | --- | --- | --- | --- | --- |
| **IRT** | **General requirements** | **C1** | | **C2** | | **C3** | | **C4** | | **C5** | | **C6** | | **C7** | | **C8** | | **C9** | | **C10** | **C11** | | **C12** |
| 1 | Was the IRT model used adequately described? |  | |  | |  | | +++ | |  | |  | |  | |  | |  | |  |  | |  |
| 2 | Was the computer software package used adequately described? |  | |  | |  | | +++ | |  | |  | |  | |  | |  | |  |  | |  |
| 3 | Was the method of estimation used adequately described? |  | |  | |  | | +++ | |  | |  | |  | |  | |  | |  |  | |  |
| 4 | Were the assumptions for estimating parameters of the IRT model checked? |  | |  | |  | | +++ | |  | |  | |  | |  | |  | |  |  | |  |
|  | **Final score Box IRT** |  | |  | |  | | **+++** | |  | |  | |  | |  | |  | |  |  | |  |
| **A** | **Internal consistency** | **C1** | | **C2** | | **C3** | | **C4** | | **C5** | | **C6** | | **C7** | | **C8** | | **C9** | | **C10** | **C11** | | **C12** |
| 1 | Does scale consist of effect indicators, i.e. is it based on a reflective model? | ok | |  | | ok | |  | | ok | |  | | ok | |  | | ok | |  | ok | | ok |
| 2 | Percentage of missing items given? | n/a | |  | | n/a | |  | | n/a | |  | | n/a | |  | | n/a | |  | n/a | | +++ |
| 3 | Description of how missing items were handled? | n/a | |  | | n/a | |  | | n/a | |  | | n/a | |  | | n/a | |  | n/a | | +++ |
| 4 | Sample size included in internal consistency analysis adequate? | +++ | |  | | +++ | |  | | ++ | |  | | +++ | |  | | +++ | |  | +++ | | ++ |
| 5 | Unidimensionality of scale checked, i.e. factor analysis or IRT model applied? | 0 | |  | | 0 | |  | | 0 | |  | | 0 | |  | | +++ | |  | 0 | | 0 |
| 6 | Sample size included in unidimensionality analysis adequate? | n/a | |  | | n/a | |  | | n/a | |  | | n/a | |  | | +++ | |  | n/a | | n/a |
| 7 | Internal consistency statistic calculated for each scale separately? | 0 | |  | | 0 | |  | | 0 | |  | | +++ | |  | | +++ | |  | 0 | | 0 |
| 8 | Any important flaws in design or method of the study? | +++ | |  | | +++ | |  | | +++ | |  | | +++ | |  | | +++ | |  | +++ | | +++ |
| 9 | For CTT, continuous scores: Cronbach's alpha calculated? | +++ | |  | | +++ | |  | | +++ | |  | | +++ | |  | | +++ | |  | 0 | | +++ |
| 10 | For CTT, dichotomous scores: Cronbach's alpha or KR-20 calculated? | n/a | |  | | n/a | |  | | n/a | |  | | n/a | |  | | n/a | |  | n/a | | n/a |
| 11 | For IRT, goodness of fit statistic at global level calculated? | n/a | |  | | n/a | |  | | n/a | |  | | n/a | |  | | n/a | |  | n/a | | n/a |
|  | **Final score Box A** | **0** | |  | | **0** | |  | | **0** | |  | | **0** | |  | | **+++** | |  | **0** | | **0** |
| **B** | **Reliability** | **C1** | | **C2** | | **C3** | | **C4** | | **C5** | | **C6** | | **C7** | | **C8** | | **C9** | | **C10** | **C11**** | | **C12** |
| 1 | Percentage of missing items given? | n/a | |  | | n/a | | n/a | |  | | n/a | | n/a | | n/a | | n/a | | n/a | n/a;n/a | | +++ |
| 2 | Description of how missing items were handled? | n/a | |  | | n/a | | n/a | |  | | n/a | | n/a | | n/a | | n/a | | n/a | n/a;n/a | | +++ |
| 3 | Sample size included in analysis adequate? | +++ | |  | | + | | +++ | |  | | +++ | | +++ | | +++ | | +++ | | 0 | 0;0 | | ++ |
| 4 | At least two measurements available? | n/a | |  | | n/a | | n/a | |  | | n/a | | n/a | | n/a | | n/a | | n/a | +++;+++ | | n/a |
| 5 | Administrations independent? | n/a | |  | | n/a | | n/a | |  | | +++ | | +++ | | n/a | | +++ | | ++ | +++;+++ | | n/a |
| 6 | Time interval stated? | n/a | |  | | n/a | | n/a | |  | | n/a | | n/a | | n/a | | n/a | | n/a | +++;n/a | | n/a |
| 7 | Patients stable in the interim period on the construct to be measured? | n/a | |  | | n/a | | n/a | |  | | n/a | | n/a | | n/a | | n/a | | n/a | n/a;n/a | | n/a |
| 8 | Time interval appropriate? | n/a | |  | | n/a | | n/a | |  | | n/a | | n/a | | n/a | | n/a | | n/a | +++;n/a | | n/a |
| 9 | Test conditions similar for both measurements? | n/a | |  | | n/a | | n/a | |  | | +++ | | +++ | | n/a | | +++ | | ++ | +++;+++ | | n/a |
| 10 | Any important flaws in design or methods of the study? | +++ | |  | | +++ | | +++ | |  | | +++ | | +++ | | +++ | | +++ | | +++ | +++;+++ | | +++ |
| 11 | For continuous scores: intraclass correlation coefficient (ICC) calculated? | +++ | |  | | n/a | | +++ | |  | | +++ | | +++ | | +++ | | +++ | | n/a | +;+ | | +++ |
| 12 | For dichotomous/nominal/ordinal scores: kappa calculated? | n/a | |  | | +++ | | n/a | |  | | n/a | | n/a | | n/a | | n/a | | +++ | n/a;n/a | | n/a |
| 13 | For ordinal scores: weighted kappa calculated? | n/a | |  | | + | | n/a | |  | | n/a | | n/a | | n/a | | n/a | | + | n/a;n/a | | n/a |
| 14 | For ordinal scores: weighting scheme described? | n/a | |  | | n/a | | n/a | |  | | n/a | | n/a | | n/a | | n/a | | n/a | n/a;n/a | | n/a |
|  | **Final score Box B** | **+++** | |  | | **+** | | **+++** | |  | | **+++** | | **+++** | | **+++** | | **+++** | | **0** | **0;0** | | **++** |
| **C** | **Measurement error** |  | |  | |  | |  | |  | |  | |  | |  | |  | |  |  |  | |
| **D** | **Content validity** | **C1** | | **C2** | | **C3** | | **C4** | | **C5** | | **C6** | | **C7** | | **C8** | | **C9** | | **C10** | **C11** | **C12** | |
| 1 | Assessment of whether all items refer to relevant aspects of the construct? | +++ | |  | | 0 | |  | | 0 | |  | |  | | +++ | | +++ | | + | 0 |  | |
| 2 | Assessment of whether all items are relevant for the study population? | 0 | |  | | 0 | |  | | 0 | |  | |  | | +++ | | 0 | | 0 | 0 |  | |
| 3 | Assessment of whether all items are relevant for the purpose of instrument? | +++ | |  | | + | |  | | + | |  | |  | | +++ | | +++ | | + | + |  | |
| 4 | Assessment of whether all items together reflect the construct? | +++ | |  | | 0 | |  | | 0 | |  | |  | | + | | +++ | | + | 0 |  | |
| 5 | Any important flaws in design or method of the study? | +++ | |  | | +++ | |  | | +++ | |  | |  | | +++ | | +++ | | +++ | +++ |  | |
|  | **Final score Box D** | **0** | |  | | **0** | |  | | **0** | |  | |  | | **+** | | **0** | | **0** | **0** |  | |
| **E** | **Structural validity** | **C1** | | **C2** | | **C3** | | **C4** | | **C5** | | **C6** | | **C7** | | **C8** | | **C9** | | **C10** | **C11** | **C12** | |
| 1 | Does scale consist of effect indicators, i.e. is it based on a reflective model? |  | | ok | |  | |  | |  | |  | |  | |  | | ok | |  |  |  | |
| 2 | Percentage of missing items given? |  | | n/a | |  | |  | |  | |  | |  | |  | | n/a | |  |  |  | |
| 3 | Description of how missing items were handled? |  | | n/a | |  | |  | |  | |  | |  | |  | | n/a | |  |  |  | |
| 4 | Sample size included in analysis adequate? |  | | +++ | |  | |  | |  | |  | |  | |  | | +++ | |  |  |  | |
| 5 | Any important flaws in design or method of the study? |  | | +++ | |  | |  | |  | |  | |  | |  | | +++ | |  |  |  | |
| 6 | For CTT: exploratory or confirmatory factor analysis performed? |  | | ++ | |  | |  | |  | |  | |  | |  | | +++ | |  |  |  | |
| 7 | For IRT: tests for determining (uni-) dimensionality of the items performed? |  | | n/a | |  | |  | |  | |  | |  | |  | | n/a | |  |  |  | |
|  | **Final score Box E** |  | | **++** | |  | |  | |  | |  | |  | |  | | **+++** | |  |  |  | |
| **F** | **Hypotheses testing** | **C1** | | **C2** | | **C3** | | **C4** | | **C5** | | **C6** | | **C7** | | **C8***** | | **C9** | | **C10** | **C11***** | **C12** | |
| 1 | Percentage of missing items given? |  | |  | | n/a | |  | | n/a | | n/a | | n/a | | n/a;n/a | | n/a | |  | n/a;n/a | +++ | |
| 2 | Description of how missing items were handled? |  | |  | | n/a | |  | | n/a | | n/a | | n/a | | n/a;n/a | | n/a | |  | n/a;n/a | +++ | |
| 3 | Sample size included in analysis adequate? |  | |  | | ++ | |  | | 0 | | ++ | | +++ | | ++;0 | | +++ | |  | 0;0 | ++ | |
| 4 | Hypotheses regarding correlations/mean differences formulated a priori? |  | |  | | +++ | |  | | + | | ++ | | +++ | | +;+++ | | ++ | |  | +;+ | + | |
| 5 | Expected direction of correlations/mean differences included in hypotheses? |  | |  | | +++ | |  | | ++ | | +++ | | +++ | | ++;+++ | | ++ | |  | +++;+++ | ++ | |
| 6 | Expected absolute/relative magnitude of correlations/mean differences included in hypotheses? |  | |  | | ++ | |  | | ++ | | ++ | | ++ | | ++;++ | | ++ | |  | ++;++ | ++ | |
| 7 | For convergent validity: adequate description provided of comparator instrument(s)? |  | |  | | n/a | |  | | n/a | | +++ | | +++ | | +++;+++ | | n/a | |  | n/a;+++ | + | |
| 8 | For convergent validity: measurement properties of comparator instrument(s) adequately described? |  | |  | | n/a | |  | | n/a | | 0 | | 0 | | 0;0 | | n/a | |  | n/a;0 | 0 | |
| 9 | Any important flaws in design or method of the study? |  | |  | | +++ | |  | | +++ | | +++ | | +++ | | +++;+++ | | +++ | |  | +++;+++ | +++ | |
| 10 | Design and statistical methods adequate for hypotheses to be tested? |  | |  | | ++ | |  | | 0 | | +++ | | ++ | | ++;++ | | ++ | |  | ++;++ | +++ | |
|  | **Final score Box F** |  | |  | | **++** | |  | | **0** | | **0** | | **0** | | **0;0** | | **++** | |  | **0;0** | **0** | |
| **G** | **Cross-cultural validity** | | **C1** | | **C2** | | **C3** | | **C4** | | **C5** | | **C6** | | **C7** | | **C8** | | **C9** | **C10** | **C11** | **C12** | |
| 1 | Percentage of missing items given? | |  | |  | |  | |  | |  | | n/a | |  | |  | |  |  |  |  | |
| 2 | Description of how missing items were handled? | |  | |  | |  | |  | |  | | n/a | |  | |  | |  |  |  |  | |
| 3 | Sample size included in analysis adequate? | |  | |  | |  | |  | |  | | n/a | |  | |  | |  |  |  |  | |
| 4 | Both the original language in which instrument was developed and language in which instrument was translated described? | |  | |  | |  | |  | |  | | +++ | |  | |  | |  |  |  |  | |
| 5 | Expertise of people involved in translation process adequately described? | |  | |  | |  | |  | |  | | + | |  | |  | |  |  |  |  | |
| 6 | Did translators work independently from each other? | |  | |  | |  | |  | |  | | +++ | |  | |  | |  |  |  |  | |
| 7 | Items translated forward and backward? | |  | |  | |  | |  | |  | | 0 | |  | |  | |  |  |  |  | |
| 8 | Adequate description of how differences between the original and translated versions were resolved? | |  | |  | |  | |  | |  | | +++ | |  | |  | |  |  |  |  | |
| 9 | Translation reviewed by a committee? | |  | |  | |  | |  | |  | | ++ | |  | |  | |  |  |  |  | |
| 10 | Instrument pre-tested (e.g. cognitive interviews) to check interpretation, cultural relevance of the translation, and ease of comprehension? | |  | |  | |  | |  | |  | | n/a | |  | |  | |  |  |  |  | |
| 11 | Sample used in the pre-test adequately described? | |  | |  | |  | |  | |  | | n/a | |  | |  | |  |  |  |  | |
| 12 | Samples similar for all characteristics except language and/or cultural background? | |  | |  | |  | |  | |  | | n/a | |  | |  | |  |  |  |  | |
| 13 | Any important flaws in design or methods of the study? | |  | |  | |  | |  | |  | | n/a | |  | |  | |  |  |  |  | |
| 14 | For CTT: confirmatory factor analysis performed? | |  | |  | |  | |  | |  | | n/a | |  | |  | |  |  |  |  | |
| 15 | For IRT: differential item function (DIF) between language groups assessed? | |  | |  | |  | |  | |  | | n/a | |  | |  | |  |  |  |  | |
|  | **Final score Box G** | |  | |  | |  | |  | |  | | **0** | |  | |  | |  |  |  |  | |
| **H** | **Criterion validity** | |  | |  | |  | |  | |  | |  | |  | |  | |  |  |  |  | |
| **I** | **Responsiveness** | |  | |  | |  | |  | |  | |  | |  | |  | |  |  |  |  | |

*Description of item content altered to fit this Table. For exact item content see COSMIN website ([46](#_ENREF_46)), **C11 reported inter-rater-reliability and intra-rater-reliability, ***C8 and C11 used more than one sample for hypotheses testing. Study IDs: C1 = Silverman et al. (2011), C2 = Edgcumbe, Silverman & Benson (2012), C3 = Fischbeck et al. (2011), C4 = Harasym, Woloschuk & Cunning (2008), C5 = Hodges & McIlroy (2003), C6 = Scheffer et al. (2008), C7 = Mortsiefer et al. (2014), C8 = Humphris & Kaney (2001), C9 = Huntley et al. (2012), C10 = Thistlethwaite (2002), C11 = Lang et al. (2004), C12 = Van Nuland et al. (2012). 4-point scale rating: +++ = excellent, ++ = good, + = fair, 0 = poor, empty space = COSMIN rating not applicable. n/a = not applicable.
